# Near‐infrared light‐responsive Nitric oxide microcarrier for multimodal tumor therapy

**DOI:** 10.1002/SMMD.20230016

**Published:** 2023-06-14

**Authors:** Danna Liang, Gaizhen Kuang, Xiang Chen, Jianhua Lu, Luoran Shang, Weijian Sun

**Affiliations:** ^1^ Department of Gastrointestinal Surgery The Second Affiliated Hospital and Yuying Children's Hospital of Wenzhou Medical University Wenzhou Zhejiang China; ^2^ Oujiang Laboratory (Zhejiang Lab for Regenerative Medicine, Vision and Brain Health) Wenzhou Institute University of Chinese Academy of Sciences Wenzhou Zhejiang China; ^3^ Zhongshan‐Xuhui Hospital and the Shanghai Key Laboratory of Medical Epigenetics, the International Co‐laboratory of Medical Epigenetics and Metabolism (Ministry of Science and Technology) Institutes of Biomedical Sciences Fudan University Shanghai China

**Keywords:** gas therapy, microcarriers, microfluidic electrospray, multimodal tumor therapy, nitric oxide

## Abstract

Nitric oxide (NO) has shown great potential in tumor therapy, and the development of a platform for precise and controllable NO release still needs to be explored. Herein, a microfluidic electrospray strategy is proposed for the fabrication of hydrogel microspheres encapsulating NO donors (S‐nitrosoglutathione, GSNO) together with black phosphorus (BP) and chemotherapeutic doxorubicin (DOX) as microcarriers for tumor therapy. Based on the excellent photothermal property of BP and thermal sensitivity of GSNO, the microcarriers exhibit a near‐infrared light (NIR)‐responsive NO release behavior. Besides, the photothermal performance of the microcarriers accelerates the release of DOX. All these contribute to the excellent tumor‐killing effect of the microcarriers by combining multiple therapeutic strategies including NO therapy, photothermal therapy, and chemotherapy. Moreover, it was demonstrated that the NIR‐responsive NO delivery microcarriers could significantly inhibit tumor growth without apparent side effects in vivo. Therefore, it is believed that the novel NIR‐responsive NO microcarriers are promising candidates in clinical tumor therapy applications.

## Graphical abstract

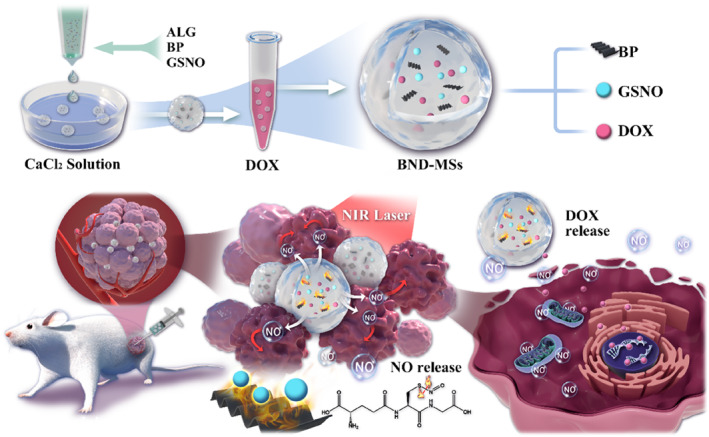

**Key points**

- A novel NIR‐responsive NO microcarrier is presented for multimodal tumor therapy.
- The microcarriers are loaded with black phosphorus (BP), NO donors (GSNO) and DOX.
- The release of NO from the microcarriers can be controlled by NIR light.
- Multimodal therapy, including NO therapy, photothermal therapy, and chemotherapy, contributes to significant tumor‐killing efficacy.

## 1 INTRODUCTION

Tumor is a thorny problem worldwide, which seriously threatens human life and health.^1^, ^2^ Different treatments, such as surgery, radiotherapy, and chemotherapy, are selected according to the stage of the tumor, among which chemotherapy is a widely used modality for many tumor types.^3^, ^4^ In general, the route of drug administration is vital for achieving optimal therapeutic efficacy.^5^, ^6^, ^7^ Compared with traditional systemic administration, localized administration by using appropriate drug carriers can not only control the release of the drug but also avoid systemic side effects.^8^, ^9^, ^10^, ^11^ In particular, microcarriers, including microspheres and microcapsules, enable efficient drug encapsulation as well as release in local areas.^12^, ^13^, ^14^ Although a lot of studies have demonstrated the value of drug microcarriers for the treatment of tumors, a large portion of them are simply loaded with chemotherapeutic agents, which still face the dilemma of toxicity, drug resistance, and restricted outcomes due to single‐modality treatment. Therefore, novel drug microcarriers for safe and effective tumor therapy are highly expected.

**FIGURE 1:**
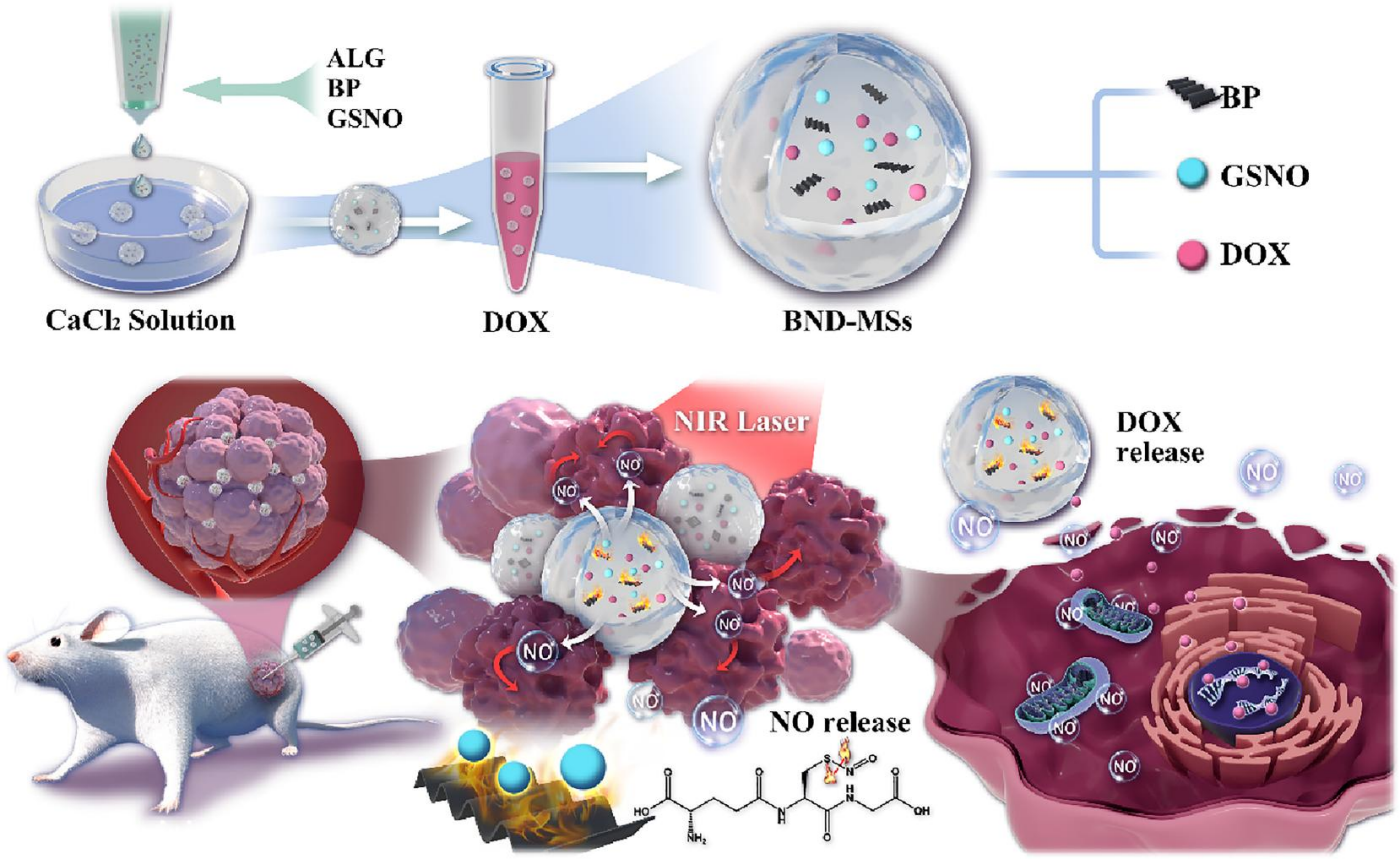
Schematic illustration of the preparation of the NIR‐responsive NO microcarriers loaded with black phosphorus (BP), NO donors (GSNO), and DOX and the localized delivery of the microcarriers for multimodal tumor therapy.

In this paper, we proposed a novel near‐infrared (NIR)‐responsive NO microcarrier for tumor therapy by a microfluidic strategy, as schemed in Figure 1. Gas therapy has recently attracted a lot of interest as a new type of therapeutic paradigm.^15^ Some gasotransmitter molecules, such as NO, carbon monoxide (CO), and hydrogen sulfide (H_2_S), can kill tumor cells at elevated concentrations, while at the same time, they are important for maintaining physiological functions.^16^, ^17^ Thus, gas therapy is considered as a “green” treatment option. Specifically, NO kills tumor cells at high concentrations (>nM) through multiple mechanisms and can synergistically improve the outcome of other treatment types.^18^, ^19^ However, accumulation and delivery of NO in tumors is a key issue in NO gas therapy, which can be solved with the use of gas‐releasing molecules in combination with proper triggering methods.^20^, ^21^, ^22^, ^23^ Microfluidics enables the generation of microparticles with precisely tunable dimensions and configurations.^24^, ^25^, ^26^, ^27^, ^28^ These microparticles could serve as drug carriers for accommodating multiple functional agents and realizing versatile responsive behaviors. It is thus conceived that by using microfluidics to fabricate microparticles containing NO donor and stimuli‐responsive materials, novel carriers for efficient NO loading and release could be constructed for tumor therapy.

Herein, we prepared NIR‐responsive NO microcarriers encapsulating S‐nitrosoglutathione (GSNO), black phosphorus (BP) and doxorubicin (DOX) for multimodal tumor therapy including NO therapy, photothermal therapy, and chemotherapy. Alginate (ALG) hydrogel microspheres were generated through microfluidic electrospray via ionic crosslinking. Benefiting from the photothermal conversion ability of BP and the S‐N bond breakage of GSNO caused by the temperature rise, the prepared microspheres displayed excellent NIR‐responsive NO release ability. In addition, DOX was loaded into the hydrogel microspheres to synergistically kill tumor cells, and its release was accelerated benefitting from the photothermal effect of the microspheres. The microspheres thus served as multifunctional microcarriers. Based on this, we confirmed through in vitro experiments that the microcarriers could remarkably enhance the apoptosis of tumor cells. We further demonstrated that the NO therapy combined with photothermal therapy and chemotherapy strategy achieved significant antitumor outcomes with minimal side effects in vivo. These results make the present microcarriers a promising platform for multimodal tumor therapy.

## 2 RESULTS

**FIGURE 2:**
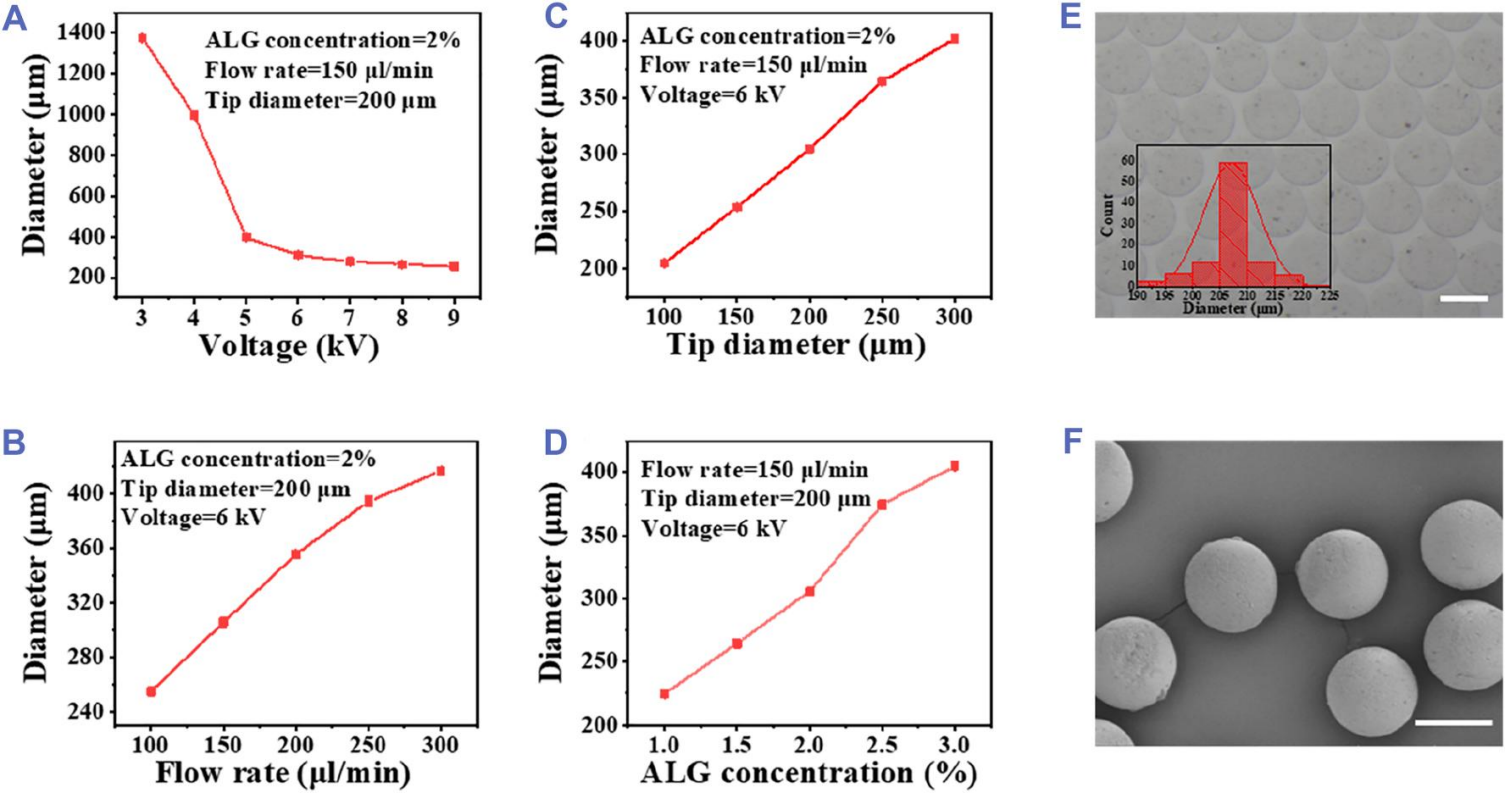
Plots of the diameter of the MSs and Characterization of B‐MSs. Plots of the diameter of the microspheres as a function of (A) vol, (B) flow rate, (C) tip diameter, and (D) ALG concentration. (E) Optical microscopic image and diameter distribution of the B‐MSs (scale bar, 200 μm). (F) SEM image of the B‐MSs (scale bar, 100 μm).

In a typical experiment, microspheres were prepared using a microfluidic chip (Figure S1A). A precursor solution of sodium alginate flowed through the device and was subjected to electrospray at the outlet of the channel (Figure S1B). Droplets were formed consecutively and then rapidly cross‐linked with calcium chloride in a container to obtain uniform and stable calcium alginate microspheres (MSs). In addition, MSs of various sizes can be prepared by adjusting different parameters, including voltage, capillary diameter, flow rate, and sodium alginate concentration. As shown in Figure 2A–D, when the voltage increased from 3 to 9 kV, the diameter decreased from 1375 to 250 μm; when the tip diameter increased from 100 to 300 μm, the diameter increased from 205 to 400 μm; when the flow rate increased from 100 to 300 μL/min, the diameter increased from 254 to 416 μm; when the concentration of sodium alginate increased from 1.0 to 3.0 wt%, the diameter increased from 225 to 405 μm. For injection into tumors, a voltage of 6 kV, tip diameter of 100 μm, sodium alginate concentration of 2%, and flow rate of 150 μL/min were selected to prepare MSs with a diameter of around 200 μm.

The microscopic image showed that the as‐prepared MSs were homogeneous and monodisperse in size (Figure S2A). Scanning electron microscopy (SEM) was used for further characterizing the microstructure of the MSs, and the results showed that the surface of the MSs is dense (Figure S2B). Besides, the swelling of the MSs reached equilibrium in 6 h, and the microspheres were completely degraded in 11 days (Figure S3A,B). These results indicated that the ALG hydrogel has good swelling property and biodegradability. Next, BP‐incorporated microspheres (B‐MSs) were prepared by pre‐mixing the dispersion of BP nanosheets with sodium alginate during the microfluidic electrospray process. The resultant B‐MSs also had a spherical shape and uniform size (Figure 2E). The surface structure of the B‐MSs was not significantly different from that of the pure ALG microspheres (Figure 2F).

**FIGURE 3:**
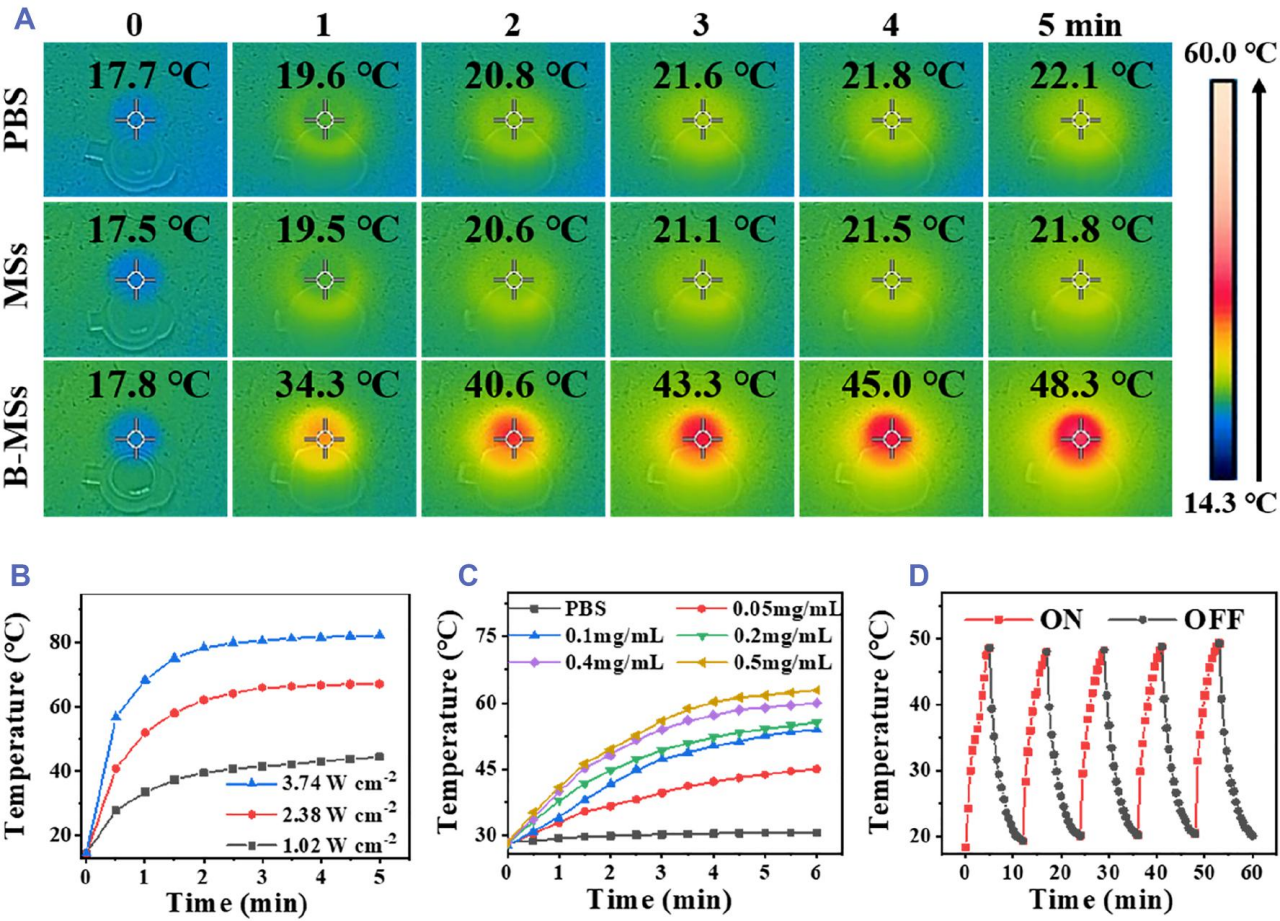
Photothermal performance of the B‐MSs. (A) Thermal images of PBS, MSs, and B‐MSs (BP concentration 0.5 mg/ml in MSs and B‐MSs) irradiated by 1 W cm^−2^ NIR for 5 min. (B) Photothermal curves of the B‐MSs with a BP concentration of 0.5 mg/ml under NIR irradiation for 5 min with different power densities. (C) Photothermal curves of B‐MSs with different concentrations of BP irradiated by NIR (1 W cm^−2^, 6 min). (D) Photothermal variation of the B‐MSs with a BP concentration of 0.5 mg/ml during five ON/OFF cycles of NIR laser (1 W cm^−2^).

Benefiting from the good NIR absorption and photothermal conversion capacity of the BP nanosheets, the prepared B‐MSs are expected to show high NIR responsiveness.^29^, ^30^, ^31^ To test this, the temperature of the B‐MSs was detected in real‐time through infrared imaging under NIR irradiation (Figure 3A). The data demonstrated that the temperature of the B‐MSs rose sharply from 17.8°C to 48.3°C, while the temperature of PBS and MSs did not increase evidently. Besides, the temperature increase in the B‐MSs could be efficiently controlled by varying the laser power density and BP concentration. As shown in Figure 3B, NIR irradiation of 3.74 W cm^−2^ can rapidly result in the rise of the temperature of the B‐MSs to 70°C within 1 min. The temperature curve in Figure 3C shows that the temperature of the B‐MSs containing 0.5 mg/ml of BPs can rise to 60°C under NIR irradiation for 6 min (1 W cm^−2^). Moreover, the photothermal heating effect did not attenuate after five ON/OFF cycles, indicating the excellent thermal stability of the B‐MSs (Figure 3D). Taken together, these results demonstrated that the B‐MSs possessed controllable and repeatable NIR response ability and can act as a photothermal switch.

**FIGURE 4:**
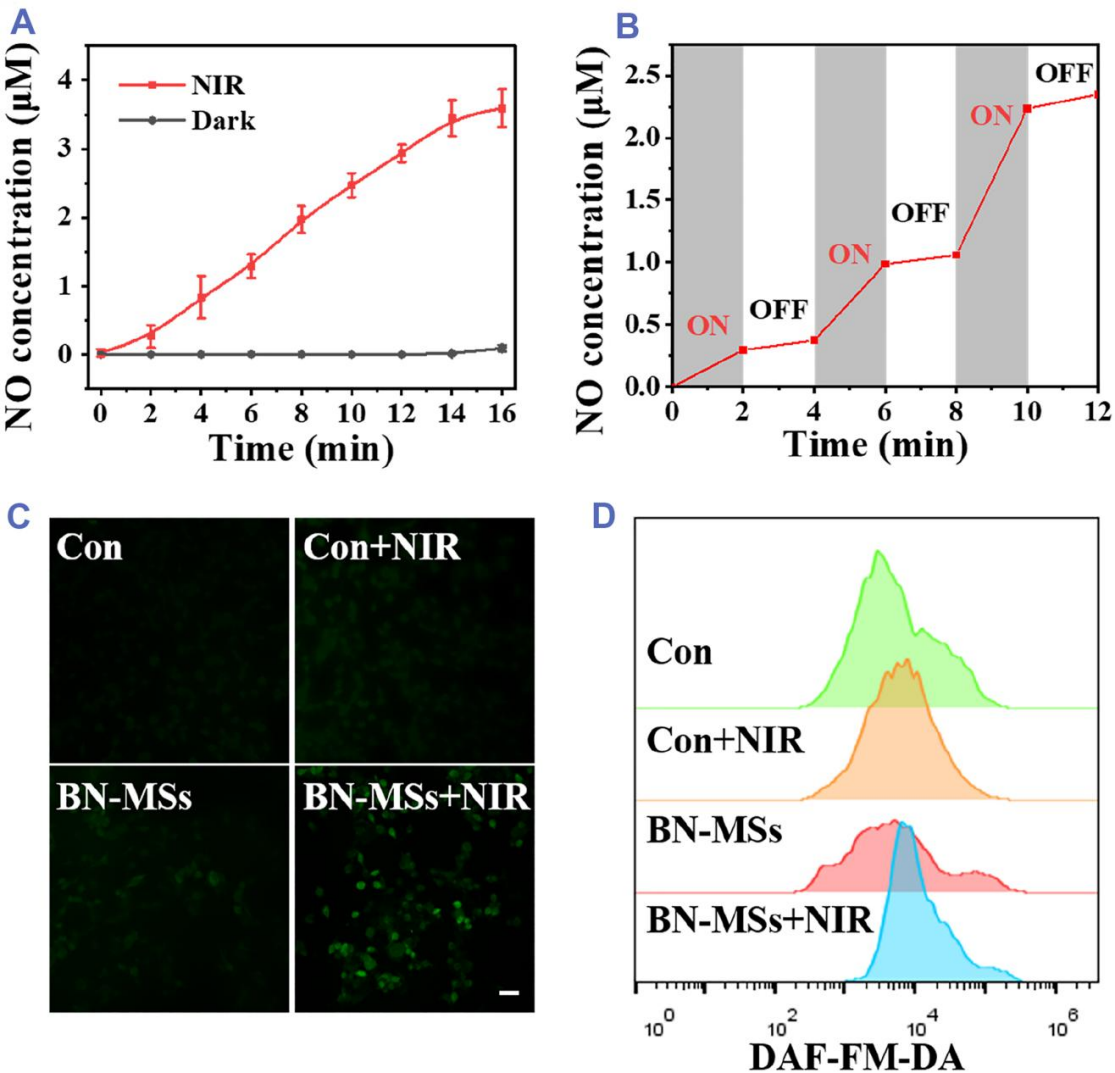
Detection of NO release from the BN‐MSs. (A) Cumulative release of NO from the BN‐MSs under NIR irradiation. (B) Release of NO under intermittent NIR irradiation. (C) Confocal images of CT26 cells stained with DAF‐FM‐DA (scale bar, 50 μm). (D) Flow cytometry detection of intracellular NO by DAF‐FM‐DA.

Based on BP's photothermal effect, we further constructed BP and GSNO‐loaded ALG microspheres (BN‐MSs) as NIR‐responsive NO delivery microcarriers. GSNO is an endogenous NO donor with low S‐N bond energy.^32^, ^33^ It has been reported that light, heat, or some metal ions could induce the S‐N bond cleavage and cause NO to be released from GSNO.^34^, ^35^, ^36^, ^37^, ^38^ Thus, the high photothermal conversion effect of BP provides a basis for the breakage of the S‐N bond. To test this, 4, 5‐diamino fluorescein (DAF‐2) was used as an indicator to detect the released NO from the BN‐MSs. When irradiated by NIR for 10 min (1 W cm^−2^), the significant increase in fluorescence in the BN‐MS group demonstrated the efficient generation of NO (Figure S4). In addition, quantitative analysis of the NO release was conducted using the classic Griess reagent. In contrast to the dark condition, the cumulative release of NO in the BN‐MS group exceeded 3.5 μM rapidly irradiated by NIR (1 W cm^−2^, 16 min) (Figure 4A). More importantly, it was found that the NO release rate had a noticeable ON/OFF‐dependence effect, indicating that NIR irradiation was the switch for controlling NO release (Figure 4B). These data demonstrated the superior NIR‐responsive NO delivery capacity of the BN‐MSs, which laid the foundation for NO therapy studies.

Subsequently, the fluorescent probe 3‐amino,4‐aminomethyl‐2′,7′‐difluorescein diacetate (DAF‐FM DA) was used to test whether NO released from the BN‐MSs could enter cells. To this end, CT26 cells stained with the DAF‐FAM DA probe were co‐cultured with BN‐MSs, and then NIR light was applied to the BN‐MSs. After 2 min of exposure to NIR (1 W cm^−2^), the formation and accumulation of intracellular NO could be tested to verify whether NO was generated in the BN‐MSs and successfully entered the cells to react with DAF‐FM and thus produce fluorescence. As observed by confocal fluorescence microscopy, CT26 cells treated with BN‐MSs exhibited bright green fluorescence under NIR irradiation, while the other groups showed very weak fluorescence (Figure 4C). The results of flow cytometry also showed that CT26 cells treated with the BN‐MSs plus NIR irradiation produced the strongest fluorescence from DAF‐FM (Figure 4D). Taken together, these results demonstrated that NO generated from the BN‐MSs upon NIR irradiation can enter cells.

**FIGURE 5:**
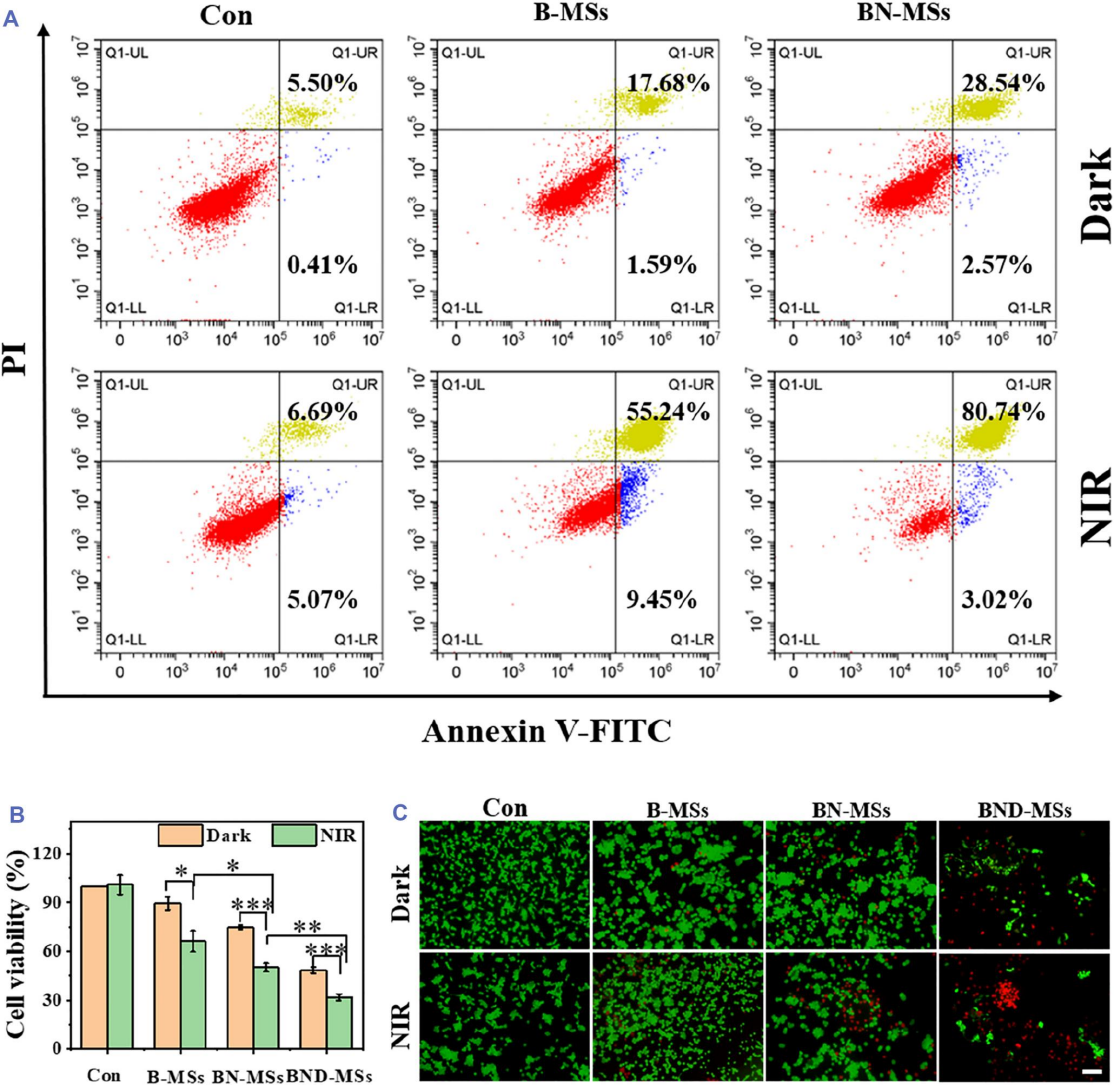
Antitumor efficiency of the BND‐MSs in vitro. (A) Flow cytometric detection of the apoptosis of CT26 cells. (B) Viability of CT26 cells in each group. (C) Fluorescent photos of CT26 cells stained with Calcein‐AM/PI (scale bar, 50 μm). (*p < 0.05, **p < 0.01).

First, the biocompatibility of the B‐MSs was evaluated by co‐culturing 3T3 cells with the leachate of microspheres that contained various concentrations of BP for 48 h. The CCK8 assay results demonstrated the negligible toxicity of the B‐MSs (Figure S5). We then explored the photothermal performance of B‐MSs in killing tumor cells under NIR irradiation. With the improvement of BP concentration and irradiation time, the proliferation of tumor cells was inhibited more obviously (Figure S6). Besides, we confirmed that free GSNO and DOX could inhibit tumor cells both in a dose‐dependent way (Figure S7 and Figure S8A). We next explored the combined effect of BP and GSNO by comparing the tumor cell killing effect of BN‐MSs (0.5 mg/ml BP and 1 mg/ml GSNO) and B‐MSs (0.5 mg/ml BP). When treated with B‐MSs plus NIR laser or BN‐MSs plus 1 W cm^−2^ NIR laser (3 min), the late apoptotic ratios of CT26 cells increased to 55.24% and 80.74%, respectively (Figure 5A), indicating that the release of NO from the BN‐MSs upon NIR irradiation could enhance the antitumor effect.

Further, we soaked BN‐MSs in a DOX solution to fabricate BND‐MSs and explored their function in tumor therapy in vitro. We first generated BP and DOX‐loaded ALG microspheres (BD‐MSs) and found that, compared with the dark condition, the photothermal conversion effect of BP greatly accelerated the release of DOX loaded in the BD‐MSs under NIR light (Figure S8B). Based on this, we expected that the BND‐MSs could exhibit multimodal therapy capacity including chemotherapy, photothermal therapy, and NO therapy. To test this, the anti‐tumor ability of B‐MSs, BN‐MSs, and BND‐MSs under NIR irradiation or dark conditions was detected by CCK8 assay. After 2 min of exposure to NIR (1 W cm^−2^), the viability of B‐MSs, BN‐MSs, or BND‐MSs‐treated cells decreased to 66%, 50%, and 31%, respectively (Figure 5B). The live/dead cell staining assay was also consistent with this result, indicating a significant antitumor effect of the multimodal therapy strategy (Figure 5C).

**FIGURE 6:**
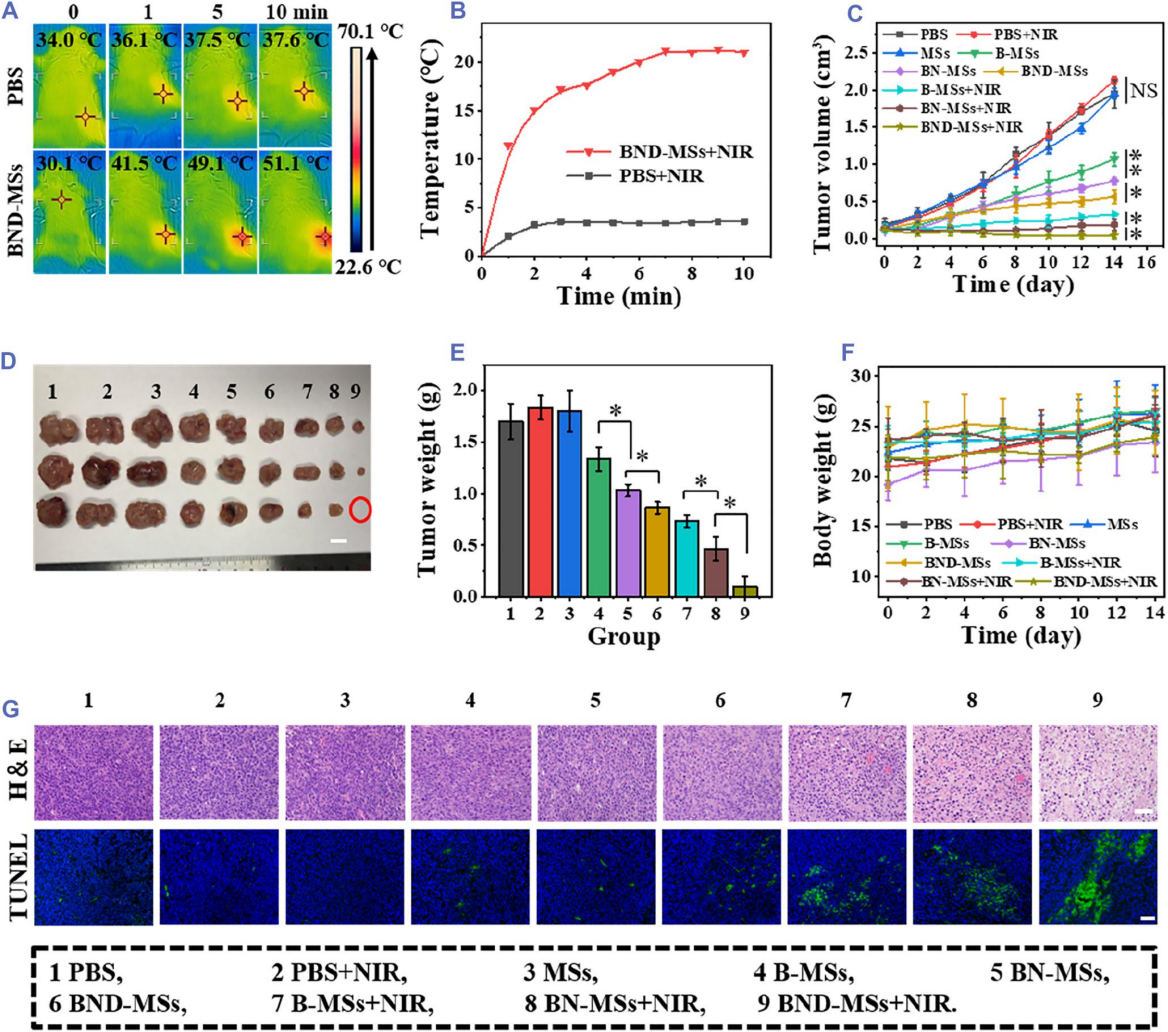
Antitumor effects of the BND‐MSs in vivo. (A) Photothermal images and (B) photothermal curves of tumors in the mice treated with PBS or BND‐MSs irradiated by 1 W cm^−2^ NIR (10 min). (C) Tumor growth curves in each group (*n* = 3). (D) Photo (scale bar, 1 cm) and (E) body weight of tumors obtained from each group on day 14 (*n* = 3). (F) Weight changes of mice in each group (*n* = 3). (G) Pathological picture of tumor tissue (scale bar, 50 μm). The tumor circled in red was completely suppressed in (d). (*p < 0.05, **p < 0.01).

Given the NIR‐responsive NO delivery and antitumor ability of the BND‐MSs microcarriers in vitro, we subsequently investigated the NIR‐promoted multimodal antitumor effect of the BND‐MSs on colon tumor bearing mice. All the mice could be separated into nine groups at random, that is, PBS, PBS + NIR, MSs, B‐MSs, BN‐MSs, BND‐MSs, B‐MSs + NIR, BN‐MSs + NIR, and BND‐MSs + NIR. Microspheres were injected into the tumor to evaluate the antitumor ability. To explore the photothermal capacity of the BND‐MSs in vivo, the temperature of the tumor was detected in real‐time via infrared imaging under NIR irradiation (Figure 6A). We controlled the temperature of PTT at about 50°C by adjusting the parameters of the NIR light. The temperature of the mice in the BND‐MS group increased by 21°C, while that in the PBS group only increased by 3.6°C after 10 min NIR irradiation, indicating the significant photothermal capacity of BND‐MSs in vivo (Figure 6B). The tumor volumes were recorded every other day. As shown in Figure 6C, tumors in the PBS, PBS + NIR, and MS groups grew rapidly, while tumor growth in the other groups was restrained to varying degrees.

We found that the B‐MSs + NIR, BN‐MSs + NIR, and BND‐MSs + NIR groups exhibited a more pronounced suppressive effect on tumors compared with the corresponding dark groups. Besides, compared with the BN‐MSs + NIR group, the B‐MSs + NIR group showed a relatively inferior antitumor effect. This indicated the role of intratumoral diffusion of NO, which promoted apoptosis of tumor cells. Of note, the most remarkable tumor suppression was observed in the BND‐MSs + NIR‐treated group, demonstrating a potent long‐term tumor suppressive effect of multimodal therapy including chemotherapy, photothermal therapy, and NO therapy. The statistical differences in tumor mass between the different groups were also consistent with the above results (Figure 6D,E). All tumor tissues were further characterized by pathological sections. Hematoxylin and eosin (H&E) staining and terminal deoxynucleotidyl transferase dUTP Nick end labeling (TUNEL) staining showed that the BND‐MSs + NIR group caused more apoptosis than the other groups (Figure 6G). The body weight of the mice was recorded every 2 days, and an overall upward trend in body weight was observed in each group (Figure 6F). H&E staining of the organs also proved that MSs, B‐MSs, BN‐MSs, and BND‐MSs had no obvious damage to the organs (Figure S9). These results confirmed the superior effect of the BND‐MSs as novel microcarriers for the multimodal treatment of tumors.

## 3 CONCLUSION

In conclusion, we have proposed a microfluidic electrospray strategy to synthesize ALG microspheres loaded with BP, GSNO, and DOX (BND‐MSs) as NO microcarriers for multimodal tumor therapy including NO therapy, PTT, and chemotherapy. The microspheres owned the advantages of high uniformity, size controllability, and biocompatibility. The addition of BP endowed the microcarriers with the performance of NIR‐responsive NO delivery, which promoted the NO‐induced apoptosis of tumor cells. Furthermore, the photothermal conversion property accelerated the release of DOX. Therefore, the use of BND‐MSs showed the most significant tumor cell killing effect compared with simple PTT with the use of B‐MSs or combined PTT and NO therapy with the use of BN‐MSs. Moreover, after localized injection of the BND‐MSs microcarriers into the tumor, the multimodal tumor therapy efficacy was demonstrated, which reflected the broad application potential of the BND‐MSs. We believe that the NO‐microcarriers could provide a new solution for clinical multimodal tumor therapy and broaden the research on NO treatment of other diseases.

## 4 EXPERIMENTAL SECTION/METHODS

*Materials*: Low viscosity sodium alginate and anhydrous calcium chloride were purchased from Alfa Aesar. Black phosphorus powder was purchased from Nanjing XFNANO Material Tech Co., LTD. DAF‐2 was purchased from Aladdin. N‐methyl‐2‐pyrrolidone (NMP), S‐nitrosoglutathione (GSNO), and doxorubicin hydrochloride were obtained from Macklin. Cell Counting Kit‐8 (CCK8), Griess reagent kit, Phosphate‐buffered saline (PBS), and DAF‐FM DA were purchased from Beyotime. Calcein AM/PI staining kit was obtained from Thermo Fisher Scientific. The apoptosis detection kit was bought from BD Biosciences.

*Synthesis of BP nanosheets*: 30 mg BP powder was dispersed in 30 ml NMP and sonicated for 12 h in an ice bath. The obtained suspension containing BP was centrifuged to remove NMP (20 min, 10,000 rpm). After that, the precipitate was rinsed repeatedly with water.

*Preparation of the BN‐MSs and BND‐MSs*: A microfluidic device was constructed for the generation of microspheres. Sodium alginate solution (2.0%, w/v), BP suspension (0.5 mg/ml), and GSNO solution (1 mg/ml) were evenly mixed as a precursor solution, and then injected into the chip via a syringe pump for electrospray. Relevant parameters were set, including the distance between the microfluidic chip and the receiving fluid (10 cm), the tip diameter (100 μm), the voltage (6 kV), the concentration of CaCl_2_ in the receiving solution (2%), and the flow rate (150 μL/min). The BN‐MSs were formed by rapid cross‐linking of calcium ions and alginate. Then, the BN‐MSs were immersed in CaCl_2_ solution for 30 min before washing twice with deionized water. Next, BND‐MSs were obtained by soaking BN‐MSs in DOX solution (1 mg/ml). Finally, the BND‐MSs were rinsed three times with DI water and freeze‐dried for later use.

*Characterization*: The morphology of the MSs was characterized via a scanning electron microscope (Hitachi, SU8010) and an ordinary light microscope (Olympus BX51). Thermal images were recorded by an infrared imager (FLIR, E5xt).

*Swelling ratio of the MSs*: 10 mg of freeze‐dried MSs was weighed and soaked in PBS at 37°C for swelling. After removing excess liquid with filter paper, the swollen MSs were weighed at the indicated time point. The swelling ratio of MS is formulated as follows: Swelling ratio = (*W*_*n*_ – *W*_*i*_)/*W*_*i*_

*W*_*n*_ refers to the weight of the swelled MSs at a certain time, and *W*_*i*_ refers to the initial weight of the MSs.

*In vitro DOX release*: 20 mg BD‐MSs were soaked in 2 ml PBS, and exposed to NIR laser or dark condition for 30 min. Every 5 min, 100 μL of supernatant was used to measure the DOX signal at a wavelength of 480 nm. And 100 μL new PBS was added to the centrifugal tubes immediately. The cumulative drug release was calculated according to the standard curve of DOX.

*Photothermal performance of the B‐MSs*: During NIR irradiation, the distance between the laser and the sample was set as 10 cm. The laser‐induced temperature increase in the microspheres with different NIR power densities (1.02, 2.38, and 3.74 W cm^−2^) and BP content (0.05, 0.1, 0.2, 0.4, and 0.5 mg/ml) was captured by an infrared imager in real‐time. The NIR laser ON/OFF cycle was performed five times.

*Detection of NO released from different microspheres*: NO was detected by DAF‐2. MSs, B‐MSs, and BN‐MSs samples were incubated under room temperature or NIR irradiation (1 W cm^−2^). The fluorescence intensity was detected by a full wavelength microplate reader. *F*_*t*_/*F*_0_ was used to determine NO release in different groups, where *F*_*t*_ is the value at the sampling time point and *F*_0_ is the value at the initial time.

*Measurement of NO release*: The NO released from the BN‐MSs was detected by a Griess reagent kit. BN‐MS samples were incubated under room temperature or NIR irradiation (1 W cm^−2^). The signal was measured at a wavelength of 540 nm. The reference standard curve for NO concentration. In addition, the NIR laser ON/OFF cycle was performed three times, and the quantification of NO at the corresponding time was the same as above.

*Intracellular NO assay using DAF‐FM*: CT26 cells could be added in 24‐well plates for 24 h. DAF‐FM solution (2 μM) was used to stain the cells for 20 min and rinsed the cells with PBS 3 times. CT26 cells were co‐cultured with the BN‐MSs for 30 min and then received dark treatment or NIR irradiation. Confocal fluorescence microscopy (Nikon, A1) was used to observe intracellular fluorescence. Flow cytometry (Beckman Coulter, CytoFLEX) was used to detect intracellular fluorescence.

*Biocompatibility of the B‐MSs*: The biocompatibility of the B‐MSs was evaluated by examining the cell viability of 3T3 cells. 3T3 cells were incubated with leachate of microspheres for 48 h. In different groups, the microspheres contained different concentrations of BP (0.05, 0.1, 0.2, 0.3, 0.4, 0.5 mg/ml). Cell viability was detected on days 1, 2, and 3 by CCK8.

*Flow cytometric detection for apoptosis*: CT26 cells could be added in 48‐well plates for 24 h. After adding B‐MSs or BN‐MSs and incubating for 2 h, NIR irradiation was performed for 3 min. After 24 h of culture, cells were treated with the Apoptosis detection kit. Then, flow cytometry was used to detect the treated cells.

*Antitumor efficiency of BND‐MS* in vitro: CT26 cells could be added in 48‐well plates for 24 h. B‐MSs, BN‐MSs, or BND‐MS were added for 2‐h incubation. After NIR irradiation, cells were cultured for 24 h. Cell viability was determined by the CCK8 kit. A fluorescence microscope (ZEISS Axio Vert. A1, Germany) was used to observe the cells stained with Calcein‐AM/PI.

*Antitumor effects of the BND‐MSs* in vivo: Male BALB/*c* mice (6–8 weeks) were subcutaneously inoculated with 1 × 10^6^ CT26 cells on the right lower limb. The volume of the tumor was measured and recorded as volume = length × (width)^2^/2. When tumor growth approached 150 mm^3^, the mice were separated into nine groups at random, i. e. PBS, PBS + NIR, MSs, B‐MSs, BN‐MSs, BND‐MSs, B‐MSs + NIR, BN‐MSs + NIR, and BND‐MSs + NIR groups. The mice were intratumorally injected with PBS containing the corresponding microcarriers (equivalent to 25 mg/kg GSNO, and 1 mg/kg DOX), and the NIR illumination time was 10 min (1 W cm^−2^) on day 0. All mouse experiments were carried out with the guidelines of the Animal Care and Use Committee and with permission from the Ethical Society for Laboratory Animal Welfare, Zhejiang Laboratory Animal Center (ZJCLA‐IACUC‐20020063). On the 14th day, the mice were euthanized, and we removed the tumor and organs to assess the therapeutic effect and biocompatible by H&E and TUNEL staining.

*Statistical Analysis*: All quantitative data were presented as means with standard deviations (*n* ≥ 3). Unpaired Student's *t*‐tests were performed for two‐group comparisons. Significant differences were considered *p < 0.05, **p < 0.01, and ***p < 0.001.

## AUTHOR CONTRIBUTIONS

Luoran Shang and Weijian Sun conceived the idea and designed the experiment; Danna Liang conducted experiments and data analysis; Gaizhen Kuang contributed to the scientific discussion of the article; Danna Liang, Xiang Chen, Jianhua Lu, Luoran Shang, and Weijian Sun wrote the manuscript.

## CONFLICT OF INTEREST STATEMENT

Luoran Shang is an executive editor for *Smart Medicine* and was not involved in the editorial review or the decision to publish this article. All authors declare that there are no competing interests.

## ETHICS STATEMENT

All mouse experiments were carried out with the guidelines of the Animal Care and Use Committee and with permission from the Ethical Society for Laboratory Animal Welfare, Zhejiang Laboratory Animal Center (ZJCLA‐IACUC‐20020063).

## Supporting information

Supporting Information S1

## References

[1] H. Sung , J. Ferlay , R. L. Siegel , M. Laversanne , I. Soerjomataram , A. Jemal , F. Bray , CA Cancer J. Clin. 2021, 71, 209. 33538338 10.3322/caac.21660

[2] S. Li , Q. Jiang , S. Liu , Y. Zhang , Y. Tian , C. Song , J. Wang , Y. Zou , G. J. Anderson , J. Han , Y. Chang , Y. Liu , C. Zhang , L. Chen , G. Zhou , G. Nie , H. Yan , B. Ding , Y. Zhao , Nat. Biotechnol. 2018, 36, 258. 29431737 10.1038/nbt.4071

[3] B. A. Chabner , T. G. Roberts , Nat. Rev. Cancer 2005, 5, 65. 15630416 10.1038/nrc1529

[4] S. Cornen , E. Vivier , Science 2018, 362, 1355. 30573614 10.1126/science.aav7871

[5] R. Xu , G. Zhang , J. Mai , X. Deng , V. Segura‐Ibarra , S. Wu , J. Shen , H. Liu , Z. Hu , L. Chen , Y. Huang , E. Koay , Y. Huang , J. Liu , J. E. Ensor , E. Blanco , X. Liu , M. Ferrari , H. Shen , Nat. Biotechnol. 2016, 34, 414. 26974511 10.1038/nbt.3506 PMC5070674

[6] C. Liang , L. Xu , G. Song , Z. Liu , Chem. Soc. Rev. 2016, 45, 6250. 27333329 10.1039/c6cs00458j

[7] K. N. Sugahara , T. Teesalu , P. P. Karmali , V. R. Kotamraju , L. Agemy , A. M. Lowy , E. Ruoslahti , J. Clin. Oncol. 2010, 28(S15), e13590.

[8] P. Mi , H. Cabral , K. Kataoka , Adv. Mater. 2020, 32, 1902604. 10.1002/adma.201902604 31353770

[9] P. Zhang , Y. Wang , J. Lian , Q. Shen , C. Wang , B. Ma , Y. Zhang , T. Xu , J. Li , Y. Shao , F. Xu , J. Zhu , Adv. Mater. 2017, 29, 1702311. 10.1002/adma.201702311 28719022

[10] J. Bariwal , H. Ma , G. A. Altenberg , H. Liang , Chem. Soc. Rev. 2022, 51, 1702. 35156110 10.1039/d1cs01074c

[11] P. Zhang , Y. Zhang , X. Ding , W. Shen , M. Li , E. Wagner , C. Xiao , X. Chen , Adv. Mater. 2020, 32, 2000013. 10.1002/adma.202000013 33035385

[12] Y. Liu , Q. Huang , J. Wang , F. Fu , J. Ren , Y. Zhao , Sci. Bull. 2017, 62, 1283. 10.1016/j.scib.2017.09.006 36659457

[13] J. Zhang , R. J. Coulston , S. T. Jones , J. Geng , O. A. Scherman , C. Abell , Science 2012, 335, 690. 22323815 10.1126/science.1215416

[14] K. D. Hermanson , D. Huemmerich , T. Scheibel , A. R. Bausch , Adv. Mater. 2007, 19, 1810.

[15] L. Chen , S. Zhou , L. Su , J. Song , ACS Nano 2019, 13, 10887. 31538764 10.1021/acsnano.9b04954

[16] L. Yu , P. Hu , Y. Chen , Adv. Mater. 2018, 30, 1801964. 10.1002/adma.201801964 30066474

[17] C. Szabo , Nat. Rev. Drug Discov. 2016, 15, 185. 26678620 10.1038/nrd.2015.1 PMC5319818

[18] Y. Wang , T. Yang , Q. He , Natl. Sci. Rev. 2020, 7, 1485. 34691545 10.1093/nsr/nwaa034 PMC8291122

[19] A. W. Carpenter , M. H. Schoenfisch , Chem. Soc. Rev. 2012, 41, 3742. 22362384 10.1039/c2cs15273h PMC3341526

[20] J. Fan , Q. He , Y. Liu , F. Zhang , X. Yang , Z. Wang , N. Lu , W. Fan , L. Lin , G. Niu , N. He , J. Song , X. Chen , ACS Appl. Mater. Interfaces 2016, 8, 13804. 27213922 10.1021/acsami.6b03737 PMC5233726

[21] X. Jia , Y. Zhang , Y. Zou , Y. Wang , D. Niu , Q. He , Z. Huang , W. Zhu , H. Tian , J. Shi , Y. Li , Adv. Mater. 2018, 30, 1704490. 10.1002/adma.201704490 29889325

[22] D. A. Riccio , M. H. Schoenfisch , Chem. Soc. Rev. 2012, 41, 3731. 22362355 10.1039/c2cs15272j PMC3341515

[23] Q. Xiang , B. Qiao , Y. Luo , J. Cao , K. Fan , X. Hu , L. Hao , Y. Cao , Q. Zhang , Z. Wang , Theranostics 2021, 11, 1953. 33408791 10.7150/thno.52997 PMC7778583

[24] L. Shang , Y. Cheng , Y. Zhao , Chem. Rev. 2017, 117, 7964. 28537383 10.1021/acs.chemrev.6b00848

[25] L. Cai , F. Bian , H. Chen , J. Guo , Y. Wang , Y. Zhao , Chem 2021, 7, 93.

[26] H. Wang , Z. Zhao , Y. Liu , C. Shao , F. Bian , Y. Zhao , Sci. Adv. 2018, 4, aat2816. 10.1126/sciadv.aat2816 PMC6003728 29922720

[27] Y. Yu , L. Shang , W. Gao , Z. Zhao , H. Wang , Y. Zhao , Angew. Chem. Int. Ed. 2017, 56, 12127. 10.1002/anie.201705667 28755398

[28] P. Zhu , L. Wang , Lab Chip 2017, 17, 34. 10.1039/c6lc01018k 27841886

[29] W. Tao , X. Zhu , X. Yu , X. Zeng , Q. Xiao , X. Zhang , X. Ji , X. Wang , J. Shi , H. Zhang , L. Mei , Adv. Mater. 2017, 29, 1603276. 10.1002/adma.201603276 PMC5205548 27797119

[30] W. Chen , J. Ouyang , H. Liu , M. Chen , K. Zeng , J. Sheng , Z. Liu , Y. Han , L. Wang , J. Li , L. Deng , Y. Liu , S. Guo , Adv. Mater. 2017, 29, 1603864. 10.1002/adma.201603864 27882622

[31] L. Chen , M. Qian , H. Jiang , Y. Zhou , Y. Du , Y. Yang , T. Huo , R. Huang , Y. Wang , Biomaterials 2020, 236, 119770. 32006702 10.1016/j.biomaterials.2020.119770

[32] S. P. Singh , J. S. Wishnok , M. Keshive , W. M. Deen , S. R. Tannenbaum , Proc. Natl. Acad. Sci. U.S.A. 1996, 93, 14428. 8962068 10.1073/pnas.93.25.14428 PMC26149

[33] J. Cheng , K. He , Z. Shen , G. Zhang , Y. Yu , J. Hu , Front. Chem. 2019, 7, 530. 31403044 10.3389/fchem.2019.00530 PMC6676249

[34] W. Fan , W. Bu , Z. Zhang , B. Shen , H. Zhang , Q. He , D. Ni , Z. Cui , K. Zhao , J. Bu , J. Du , J. Liu , J. Shi , Angew. Chem. Int. Ed. 2015, 54, 14026. 10.1002/anie.201504536 26228648

[35] T. Du , Z. Qin , Y. Zheng , H. Jiang , Y. Weizmann , X. Wang , Chem 2019, 5, 2942.

[36] B. Li , P. Ji , S. Peng , P. Pan , D. Zheng , C. Li , Y. Sun , X. Zhang , Adv. Mater. 2020, 32, 2000376. 10.1002/adma.202000376 32134530

[37] R. Guo , Y. Tian , Y. Wang , W. Yang , Adv. Funct. Mater. 2017, 27, 1606398.

[38] S. Yao , M. Zheng , Z. Wang , Y. Zhao , S. Wang , Z. Liu , Z. Li , Y. Guan , Z. Wang , L. Li , Adv. Mater. 2022, 34, 2205881. 10.1002/adma.202205881 36189858

