# Supplementary material for: Near‐infrared light‐responsive Nitric oxide microcarrier for multimodal tumor therapy
Source: Smart Med. 2023 Jun 14;2(3):e20230016. doi: 10.1002/SMMD.20230016 (PMC11236066; doi:10.1002/SMMD.20230016)
Supplement: Supplementary file 1 — Supporting Information S1 [file SMMD-2-e20230016-s001.docx]

Near-infrared (NIR)-responsive NO microcarrier are fabricated for tumor therapy by microfluidic strategy. Based on the photothermal property of BP and thermal sensitivity of GSNO, the microcarriers exhibit NIR-responsive NO release and DOX accelerated release behaviors. All these contribute to the excellent tumor-killing effect both *in vitro* and *in vivo.*

Danna Liang, Gaizhen Kuang, Xiang Chen, Jianhua Lu, Luoran Shang*, Weijian Sun*

NIR-responsive NO microcarrier for multimodal tumor therapy


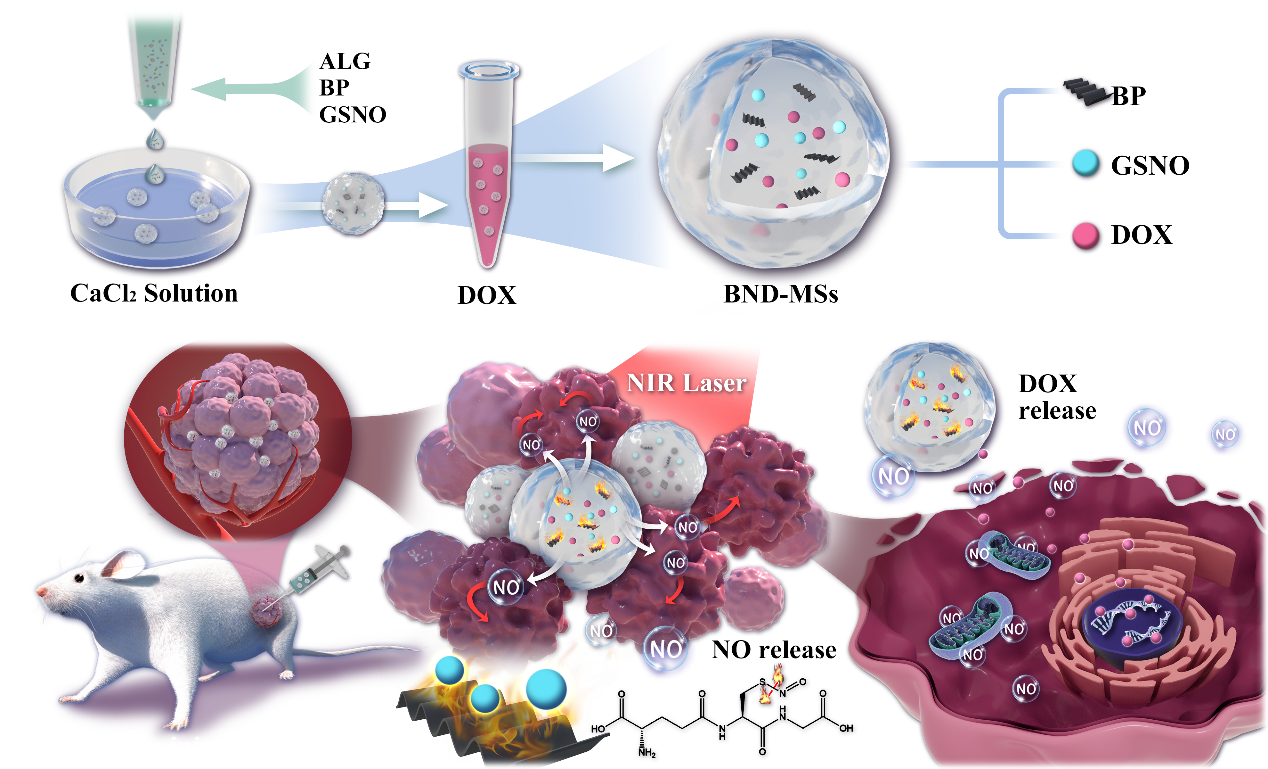


Supporting Information

NIR-responsive NO microcarrier for multimodal tumor therapy

Danna Liang, Gaizhen Kuang, Xiang Chen, Jianhua Lu, Luoran Shang*, Weijian Sun*


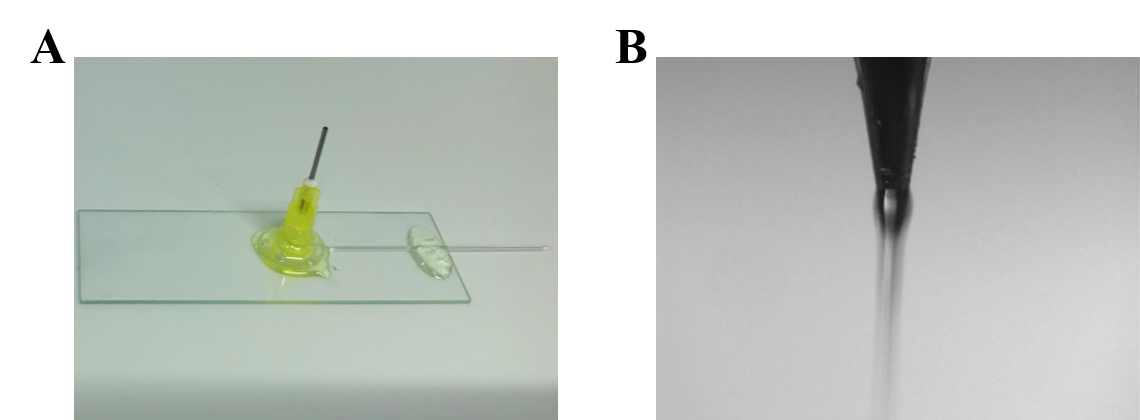


**Figure S1.** (A) Photograph of the capillary microfluidic device. (B) The formation of droplets via microfluidic electrospray.


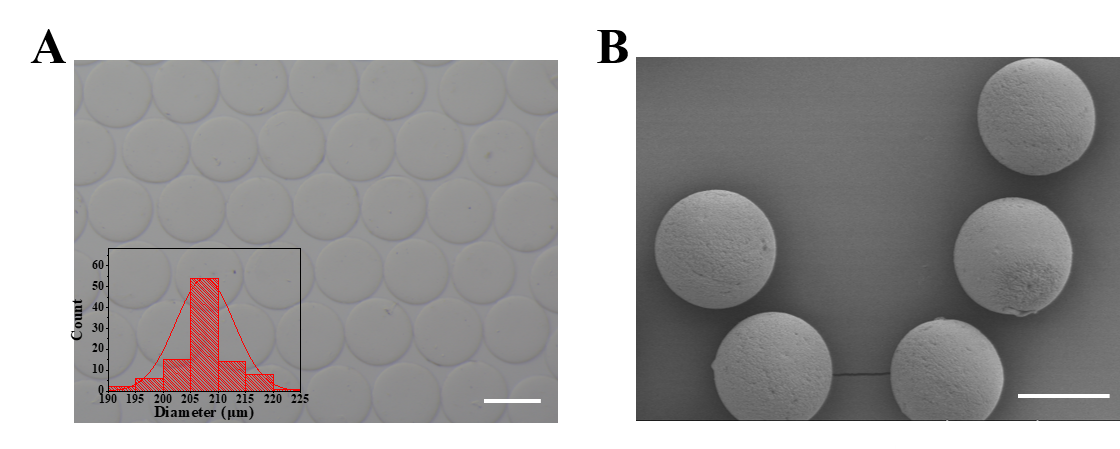


Figure S2. Characterization of MSs. (A) Optical microscopic image and diameter distribution of the MSs (scale bar, 200 μm). (B) SEM image of the MSs (scale bar, 100 μm).


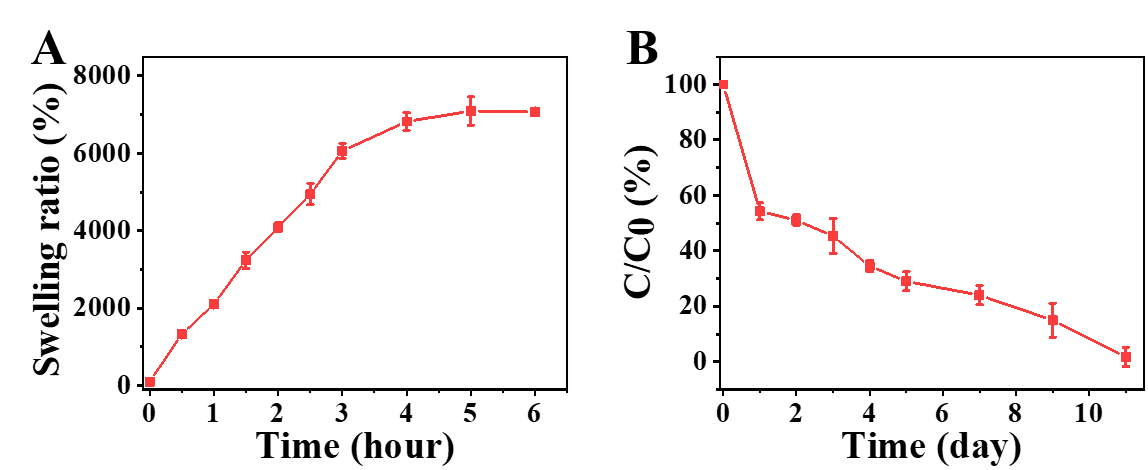


Figure S3. (A) Swelling ratio of the microspheres in PBS solution. (B) Degradation ratio of the microspheres in PBS solution.


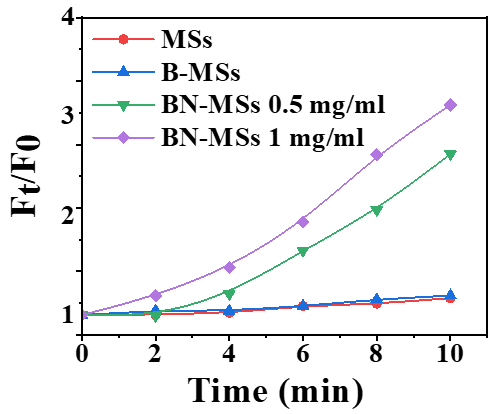


Figure S4. Release of NO in different groups (MSs, B-MSs, BN-MSs (GSNO: 0.5 mg/ml), BN-MSs (GSNO: 1 mg/ml). *F_t_* is the value at a measured time point and *F_0_* is the value at the initial time.

Figure S5. Viability of 3T3 cells co-cultured with the leachate of B-MSs contained various concentrations of BP (0.05, 0.1, 0.2, 0.3, 0.4, and 0.5 mg/mL). The cell viability of the control group was 100%, and the cell viability of each group was based on the control of each day.


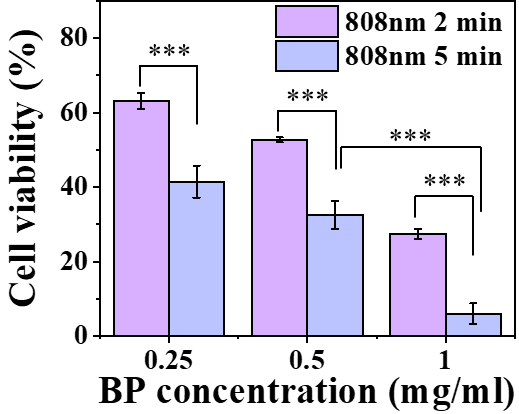


Figure S6. Viability of CT26 cells co-cultured with B-MSs contained various concentrations of BP and irradiated with NIR for different time. (***p < 0.001)


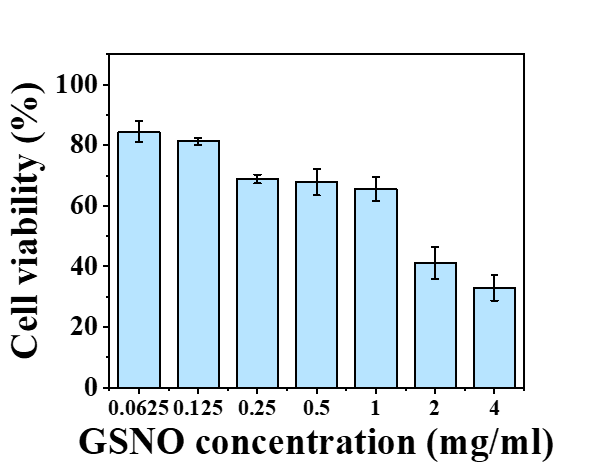


Figure S7. Viability of CT26 cells co-cultured with free GSNO.


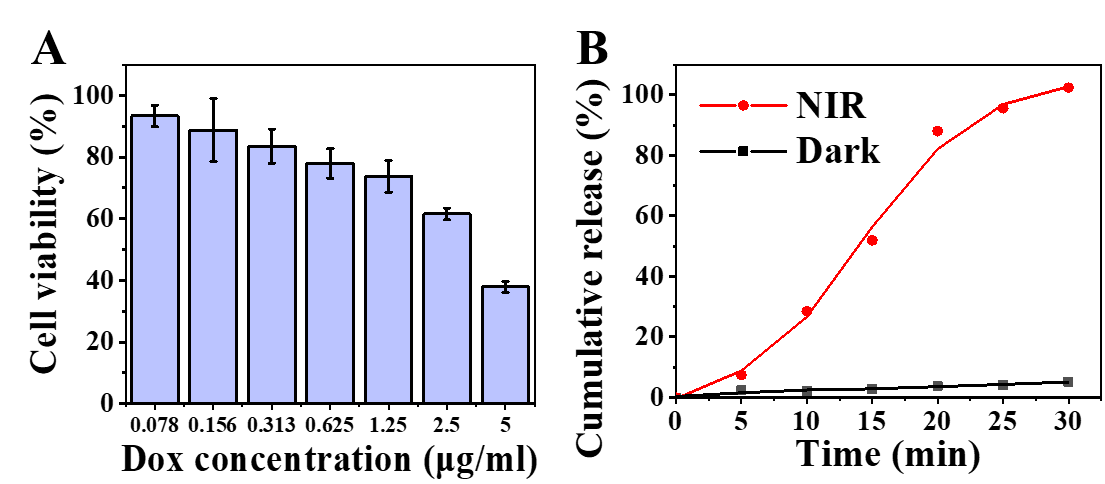


Figure S8. (A) Viability of CT26 cells co-cultured with free DOX. (B) Cumulative release of DOX from BD-MSs with or without NIR irradiation.


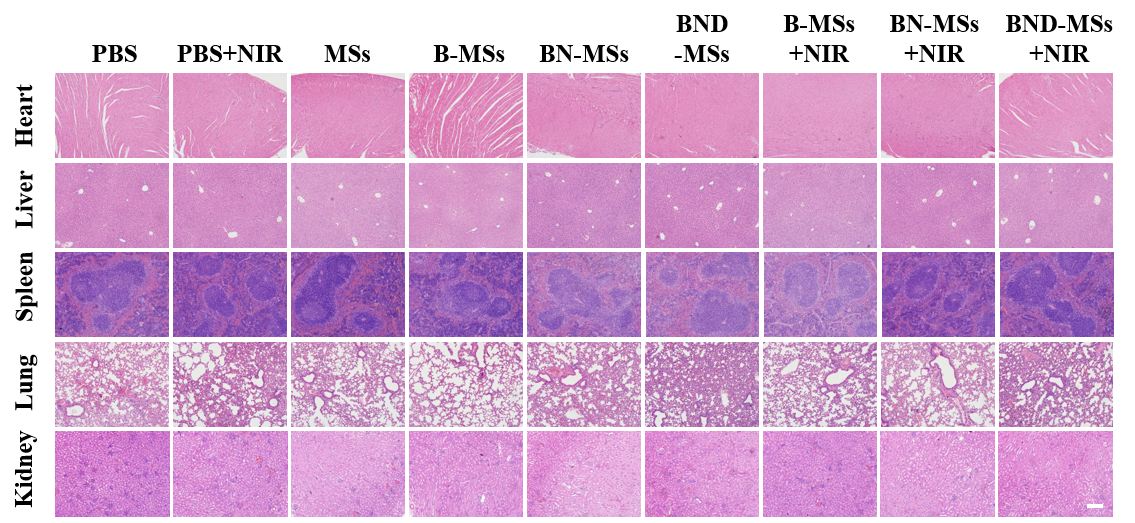


Figure S9. Characterization of organ tissues via H&E staining. Scale bar is 200 μm.
